# Clinical Utility of Transillumination on Transthoracic Imaging

**DOI:** 10.1016/j.case.2022.06.013

**Published:** 2022-08-26

**Authors:** Juan I. Cotella, Megan Yamat, Michael P. Henry, Karima Addetia, Roberto M. Lang

**Affiliations:** Department of Medicine, Section of Cardiology, University of Chicago Medical Center, Chicago, Illinois

**Keywords:** Transthoracic echocardiography, 3D echocardiography, Transillumination

## Abstract

• TI is a 3D rendering tool that applies a movable virtual light.
• TI may improve anatomic definition and diagnostic yield of transthoracic imaging.
• TI is particularly useful for optimal valve anatomy visualization.
• New 3D techniques may help achieve TTE images with similar diagnostic yield to TEE.

## Graphical abstract

**Figure:**
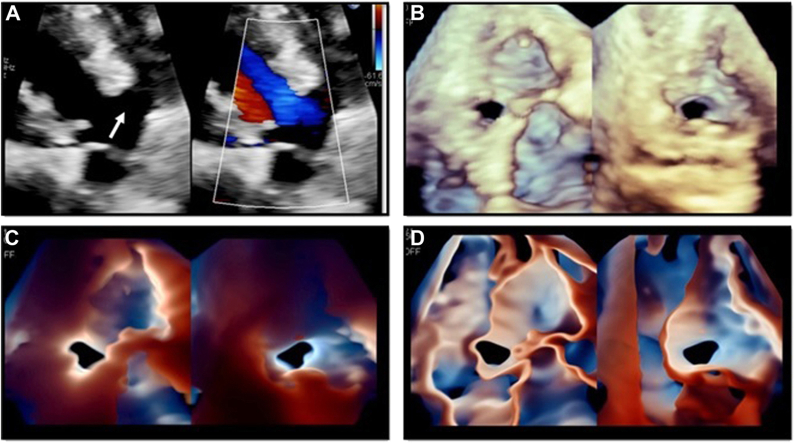

## Introduction

Transillumination (TI) is a new three-dimensional (3D) rendering tool that allows the reader to introduce a movable virtual light into a 3D data set, creating different background colors and shadow hues with the goal of enhancing the contrast between structures and improving the perception of depth and visualization of anatomic details.^1^

Traditionally, the use of TI was mostly limited to transesophageal echocardiography (TEE), where it increased the diagnostic confidence among readers.^2^ However, recent innovations in transducer technology, such as better thermal performance, wider bandwidth, wider dynamic range, and computational technology, have extended the use of this rendering technique into transthoracic echocardiography (TTE) imaging.^3^

In this case series, we aimed to illustrate the growing role and utility of TI in 3D-TTE imaging, providing examples where its use helped us to increase our diagnostic capabilities and confidence in the diagnosis.

## Case Presentation 1: Utility of TI in a Patient With a Ventricular Septal Defect

**Figure 1:**
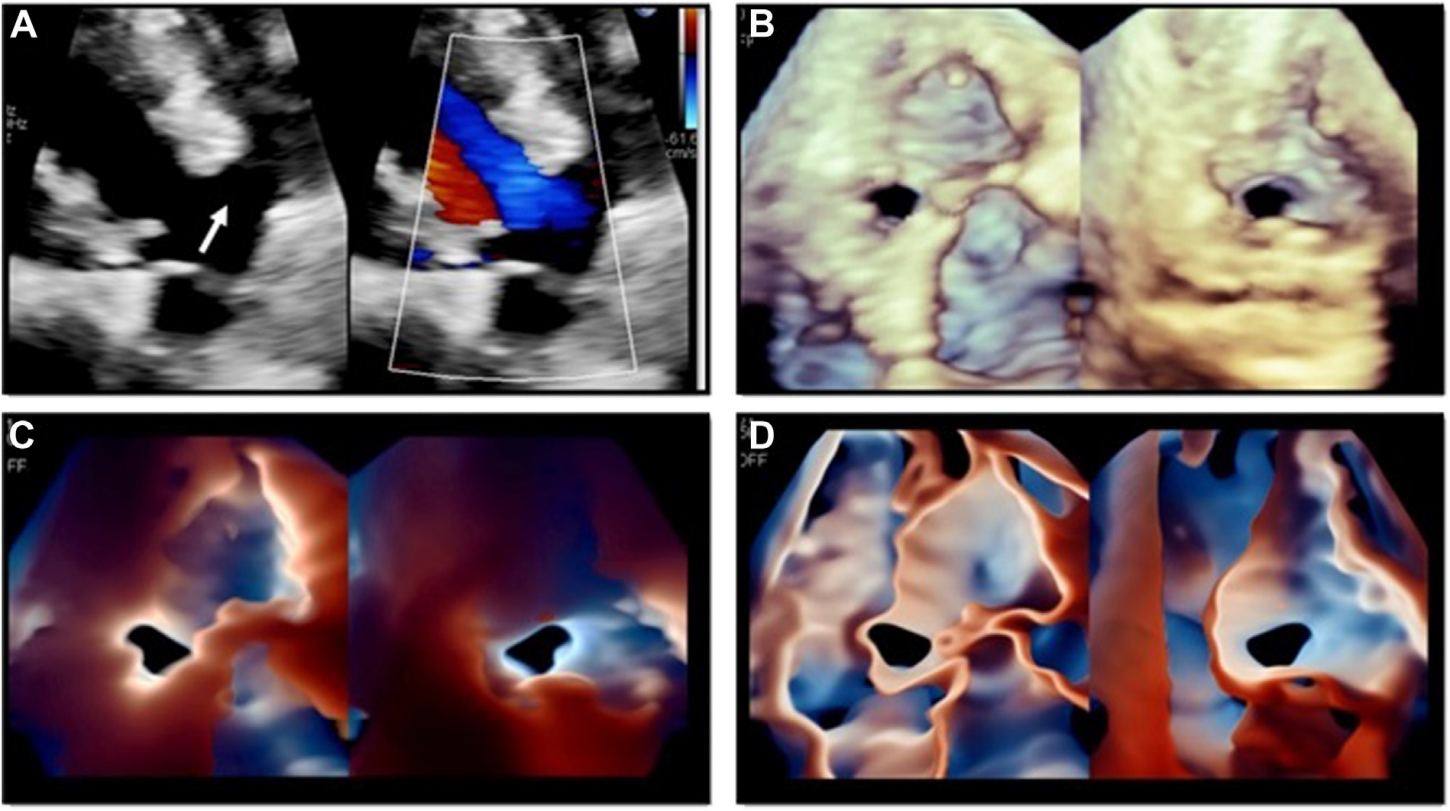
Use of TI in a patient with a diagnosis of membranous VSD. Panel A corresponds to an off-axis TTE parasternal long-axis view where a large membranous VSD can be noticed *(left image, white arrow)*. The *right image* on panel A corresponds to the use of color Doppler in this same view. Panel B corresponds to a 3D full-volume acquisition showing the precise location of the VSD (as visualized from the left ventricle *[left image]* and from the right ventricle *[right image]*). Enhanced border definition of the VSD using TI and Glass renderings are shown in panels C and D, respectively, as seen from the left ventricle *(left images)* and from the right ventricle *(right images)* as well.

A 64-year-old woman with a medical history of ventricular septal defect (VSD) presented with increasing dyspnea on exertion. A two-dimensional (2D) TTE was performed and showed a flattened interventricular septum with right ventricular (RV) enlargement and hypertrophy consistent with RV pressure and volume overload, as well as the presence of a large membranous VSD (Figure 1A). Three-dimensional full-volume acquisition shows the precise location of the defect and its relationship with adjacent structures (Figure 1B). As shown in Figure 1C and D and Video 1, the use of TI enhances the visualization of the borders and shape of the defect, which was crucial to plan and perform the percutaneous closure of the defect using an AmplatzerTM occluder.

## Case Presentation 2: Optimized Visualization of Pulmonary Valve Anatomy With TI

**Figure 2:**
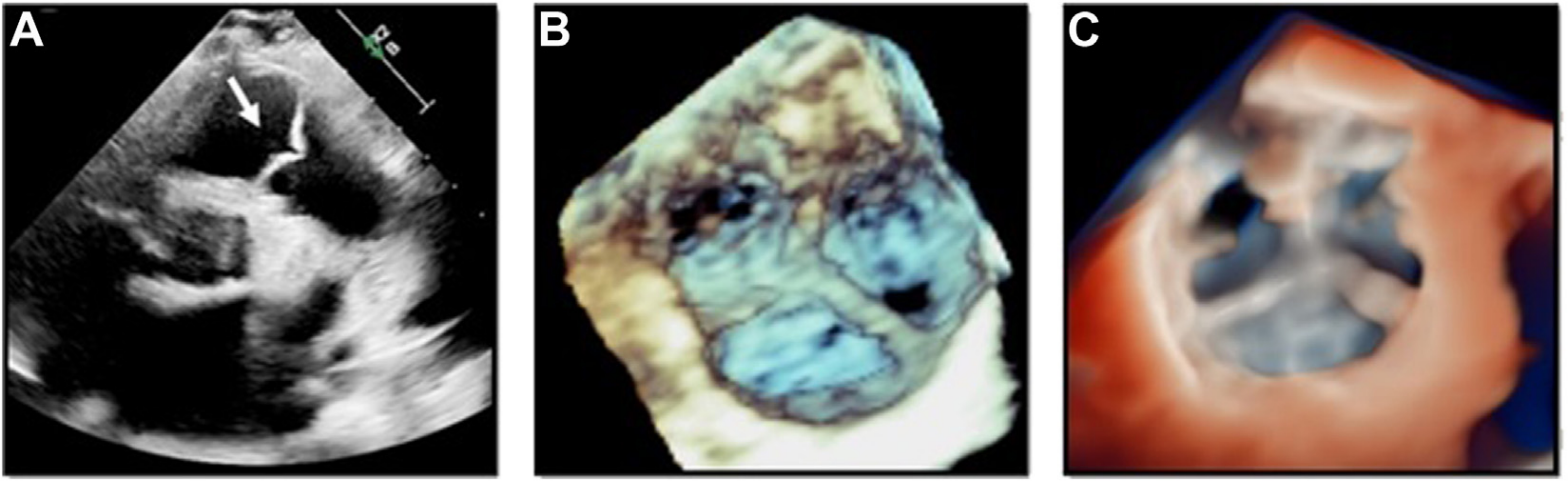
Optimized visualization of PV anatomy with TI. Panel A shows the PV seen on the PSAX view on 2D-TTE *(white arrow)*. Panel B shows a 3D zoom rendering of the PV, as seen from the RV outflow tract. From this same perspective, panel C shows increased leaflet definition and sharper commissural border delineation using TI rendering of the PV.

A 44-year-old woman with a history of heart failure with reduced ejection fraction due to amyloidosis and mild to moderate pulmonary hypertension underwent a TTE. Severe left ventricular (LV) hypertrophy and global LV systolic dysfunction (LV ejection fraction = 25%) were noted. Severe mitral regurgitation, moderately reduced RV systolic function, and mild to moderate pulmonary regurgitation were also noticed. Further assessment of the pulmonary valve (PV) was performed. Figure 2A shows the PV seen on the parasternal short-axis (PSAX) view on 2D-TTE. This case illustrates the role of 3D-TTE combined with TI to obtain an enhanced anatomic visualization of all 3 PV leaflets. Three-dimensional zoom rendering of the PV allows the reader to identify the PV valve leaflets (Figure 2B). The use of TI rendering (Figure 2C, Video 2) was able to provide clearer visualization of PV anatomy, allowing the reader to rule out the presence of structural PV disease in the context of an infiltrative cardiomyopathy.

## Case Presentation 3: Optimized Visualization of Tricuspid Valve Anatomy With TI

**Figure 3:**
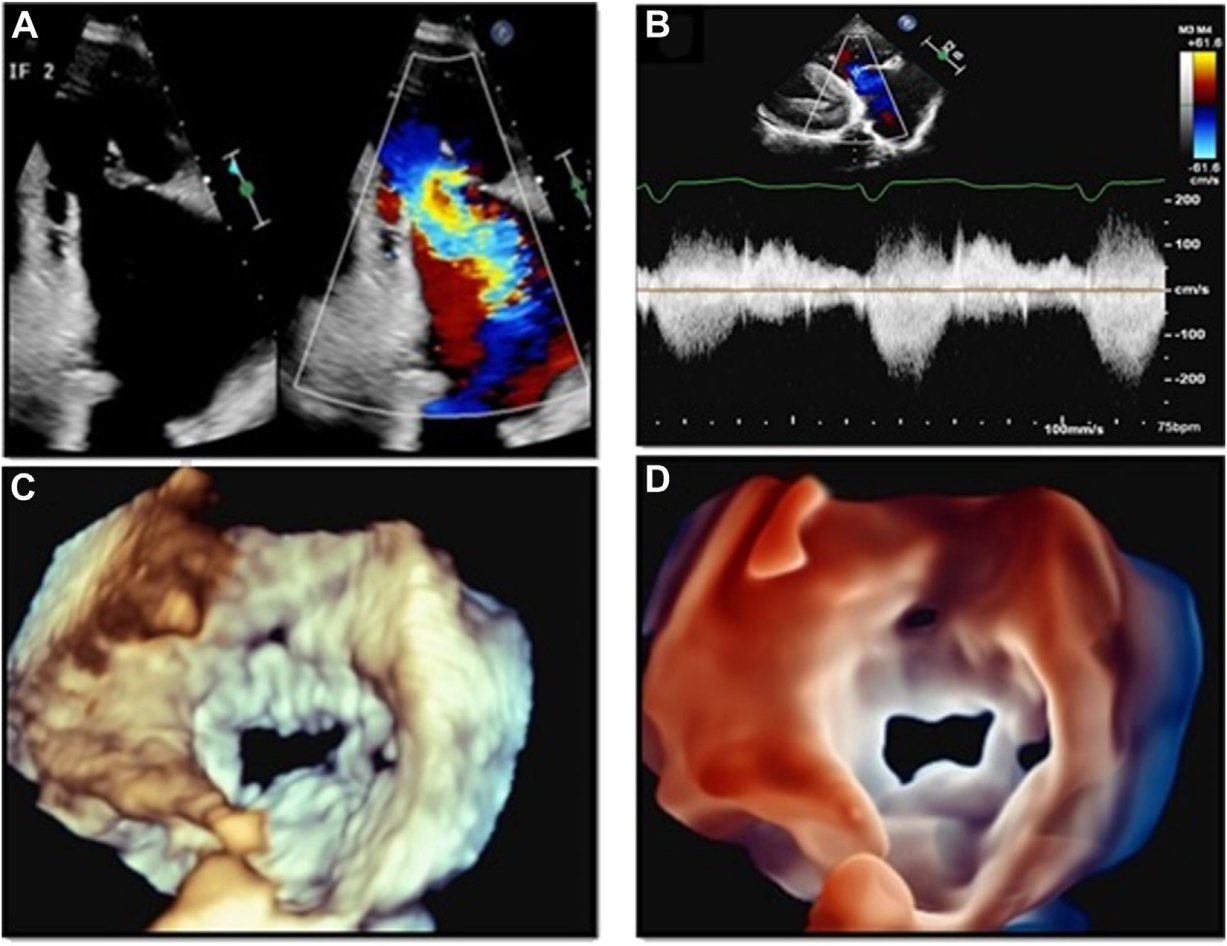
Optimized visualization of TV anatomy with TI. Panel A shows the presence of a midsystolic malcoaptation gap of the TV *(left image)* and massive TR evidenced by color Doppler *(right image)*. Panel B shows a midsystolic triangular-shaped CW Doppler tracing of the TR. Panel C shows a 3D zoom rendering of the TV as seen from the right ventricle, depicting the presence of 4 TV leaflets. Panel D shows a TTE TI rendering of the TV from the RV perspective.

A 78-year-old woman with a medical history of transthyretin amyloidosis presented for cardiac evaluation. A TTE was performed, showing a large malcoaptation gap of the tricuspid valve (TV) leaflets (Figure 3A), which were also thickened and severely tethered, resulting in massive tricuspid regurgitation (TR; Figure 3A). The presence of a tricuspid midsystolic peaking triangular-shaped flow on continuous wave (CW) Doppler (Figure 3B) was consistent with severe TR. This case reinforces the relevant role of 3D-TTE in the anatomic assessment of the TV. Three-dimensional zoom rendering of the TV depicted the presence of 4 TV leaflets at midsystole (Figure 3C). Compared to this, TI rendering allows increased perception of depth, as well as the visualization of the TV leaflets edges, with better delineation of the borders, shape, and size of the coaptation defect (Figure 3D, Video 3). The optimal visualization of the TV anatomy on this view is important, as it provides valuable information for identifying anatomic variants of the TV and information regarding edge-to-edge TV percutaneous procedure planning.

## Case Presentation 4: TI Rendering in a Patient With Aortic Valve Perforation

**Figure 4:**
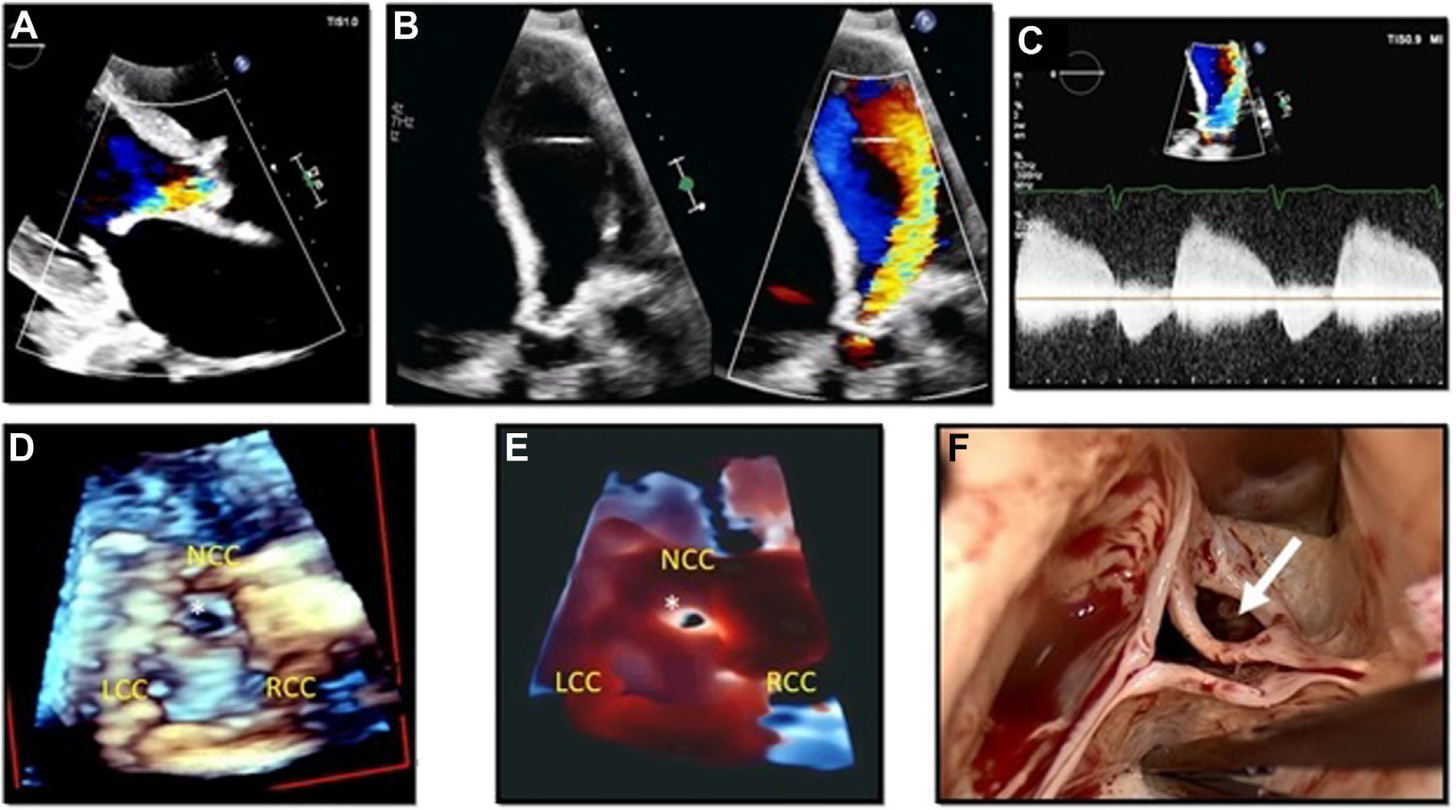
Visualization of a perforation on the AV NCC using 3D TI rendering on TTE. Panel A corresponds to a TTE PLAX view, where thickened AV leaflets and an eccentric jet of AR can be appreciated. Panel B shows thickened AV leaflets in a TTE apical 5-chamber view *(left image)*, showing with color Doppler *(right image)* the presence of a broad jet of severe AR directed toward the inferolateral LV wall. Panel C corresponds to an AV CW Doppler consistent with severe AR. Panel D shows a 3D zoom rendering of the AV. There is a defect on the NCC *(white asterisk)*, which is better depicted with the use of TI rendering as shown in panel E. Panel F corresponds to the surgical findings, showing the exact correlation with the TTE findings *(white arrow)*. *LCC*, Left coronary cusp; *RCC*, right coronary cusp.

A 69-year-old man with a history of hypertension and diabetes was hospitalized due to progressive shortness of breath and bilateral pedal edema. Eight months ago the patient was admitted with the diagnosis of aortic valve (AV) endocarditis, which was treated with intravenous antibiotics. Images obtained from a TTE in the PLAX view (Figure 4A) depict the presence of thickened AV leaflets with an eccentrically directed jet of severe aortic regurgitation (AR). An apical 5-chamber view (Figure 4B) shows that the broad jet of severe AR was directed toward the inferolateral LV wall. Figure 4C shows the corresponding AV CW Doppler tracing, which was consistent with the previous findings, although the mechanism responsible for the AR remained unclear. Figure 4D corresponds to a 3D zoom rendering of the AV showing the presence of a large perforation on the noncoronary cusp (NCC), which was likely related to the history of healed AV endocarditis. This finding is better shown using a TI rendering technique, because it increases the perception of depth and enhances the delineation of the defect borders (Figure 4E). Figure 4F shows the surgical findings and their correlation with the 3D-TTE images (Video 4).

## Case Presentation 5: Improved Delineation of a Stenotic Mitral Valve Orifice With TI Rendering

**Figure 5:**
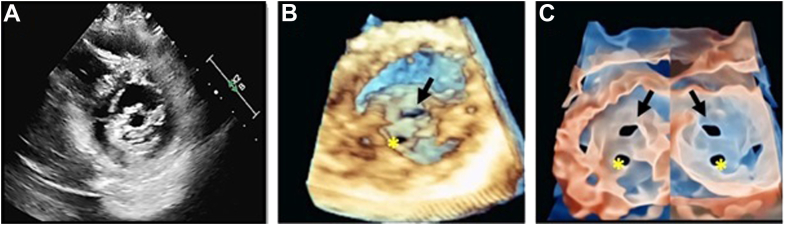
Improved MV orifice delineation with TI rendering. Panel A corresponds to a PSAX view at the level of the MV, showing bileaflet thickening and the typical fish-mouth morphology of rheumatic mitral stenosis. Panel B shows a 3D-TTE zoom rendering of the MV as seen from the LA, where a stenotic MV orifice *(black arrow)* can be seen. Please note that the second hole *(yellow asterisk)* corresponds to a dropout artifact and is not a part of the actual stenotic MV orifice. Panel C corresponds to a TTE TI rendering of the MV, where enhanced MV orifice border definition and increased perception of depth can be appreciated, both from the LA *(left image)* and the left ventricle *(right image)* perspectives.

A 47-year-old man complained of progressive shortness of breath and palpitations. A 2D-TTE PSAX view of the mitral valve (MV) shows the typical “fish-mouth” appearance of rheumatic MV stenosis (Figure 5A). Biventricular systolic function was normal, and the patient was in sinus rhythm. Mean pressure gradient across the MV was 5.6 mm Hg at a heart rate of 68 bpm. Mitral valve area calculated by planimetry was 1.3 cm^2^. These findings were consistent with moderate mitral stenosis. Three-dimensional zoom rendering of the MV (seen from the left atrium [LA]), showed a restricted opening (Figure 5B). Figure 5C and Video 5 correspond to TI renderings of the MV, as seen from the LA and from the left ventricle. This technique allows the reader to obtain a better delineation of the MV orifice, due to the improved visualization of the edges of the orifice.

## Case Presentation 6: Enhanced Definition of an MV P2 Flail Segment With the Use of 3D Transthoracic TI Rendering

**Figure 6:**
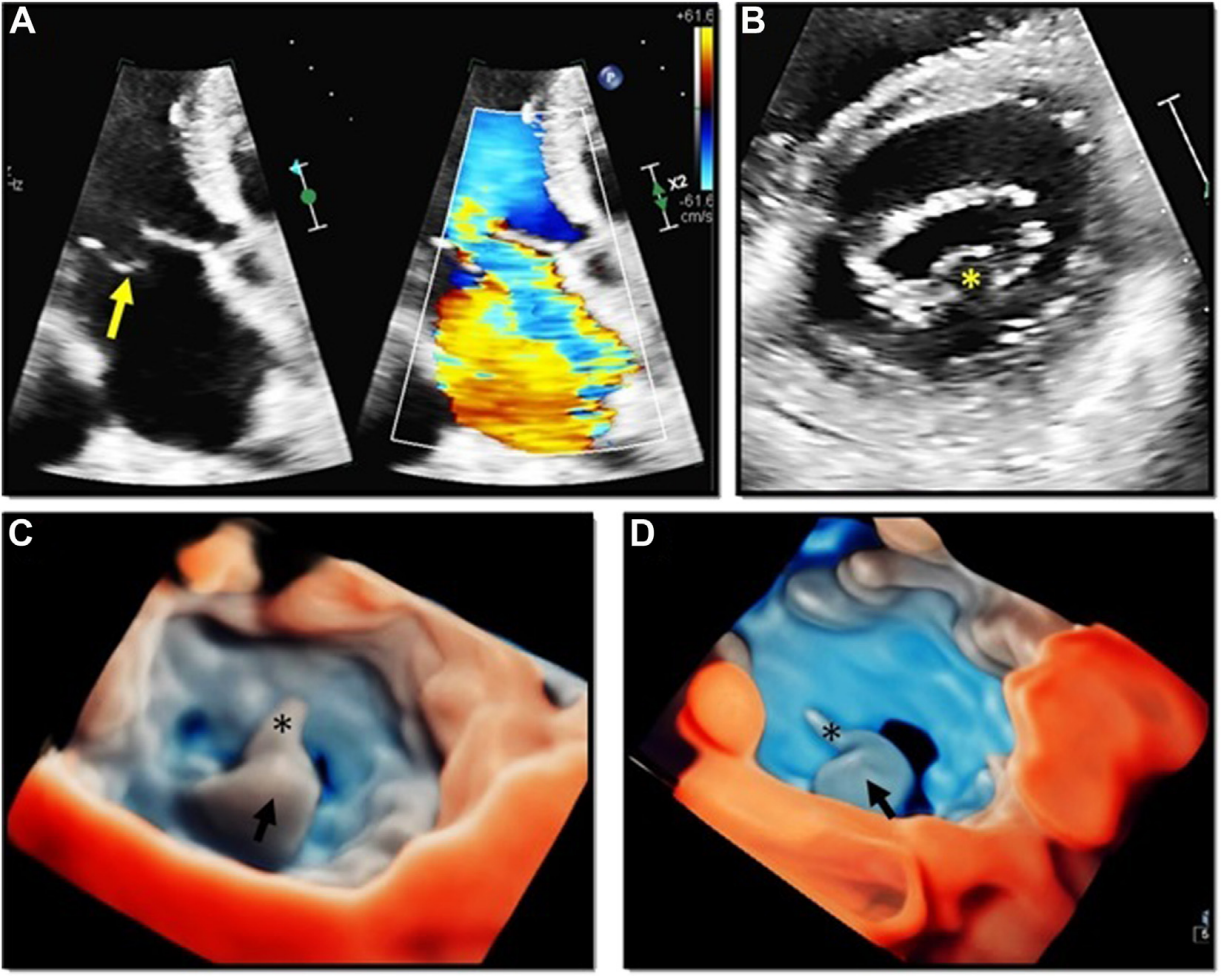
Enhanced definition of a MV P2 flail segment with the use of 3D-TTE imaging and TI rendering. Panel A shows a 2D-TTE apical 4-chamber view where a flail posterior leaflet *(left image, yellow arrow)* with a severe MR jet directed to the LA anterior wall *(right image)* can be appreciated. Panel B corresponds to a PSAX view, where the presence of a P2 flail segment was noticed *(yellow asterisk)*. Panels C and D show the advantages of 3D-TTE TI rendering, which provides optimal visualization of the flail P2 segment *(black arrow)* and the ruptured chordae *(black asterisk)* on an en face view of the MV.

A 70-year-old woman with a medical history of severe MR was referred for echocardiographic assessment of MV disease. Figure 6A shows a 2D-TTE apical 4-chamber view with a flail posterior leaflet with a severe MR jet directed to the LA anterior wall. A PSAX view corroborated the presence of a P2 flail segment (Figure 6B). The use of 3D-TTE rendering (Figure 6C) allows better visualization of the flail P2 segment and the ruptured chordae as seen from the LA. Figure 6D and Video 6 illustrate how the use or TI accentuates the definition of the affected MV scallop and the ruptured chordae, while demonstrating the delineation of the gap boundaries as well as the perception of depth in an en face view of the MV.

## Discussion

Transillumination is a 3D rendering tool that allows the introduction of a movable virtual light into a 3D data set, thereby creating different background colors and shadow hues, with the goal of enhancing the contrast between structures. In this case series, we provide examples where the diagnostic value of using TI in transthoracic studies is highlighted.

As shown in the presented cases, anatomic features enhanced by these techniques include orifice area and borders (cases 1, 3, 5, and 6), valve leaflets and edges delineation (cases 2 and 3), and structural valve disease (cases 4 and 6). As shown in case 4, the acquisition of 3D-TTE images and particularly the use of TI rendering were crucial to understand the mechanism of valve disease. Furthermore, according to the recent report by Hahn *et al.*,^4^ the use of 3D renderings in TTE allowed identification of the presence of a quadricuspid TV, as shown in case 3. These cases showed that the use of TI in TTE studies might help to provide image quality comparable to TEE imaging.

Other scenarios where the application of TI on TTE images could provide important additional information include the evaluation of thromboembolic sources, identification of vegetations^5^ and prosthetic valve dehiscence,^6^ and guidance of transcatheter procedures.^7^

## Conclusion

In the current era of structural heart disease, a thoughtful understanding and application of these tools in both TTE and TEE is important for better anatomical assessment, adding value to conventional 3D-TTE and possibly reducing the need for TEE in specific circumstances.

## Supplementary Data

Video 1 Ventricular septal defect as seen from the **(A)** LV and **(B)** RV using TTE TI to increase the sharpness and definition of the borders of the defect. *LV*, Left ventricle; *RV*, right ventricle*.*

Video 2 **(A)** Three-dimensional TTE zoom acquisition of the PV. **(B)** Visualization of the PV using TI provides increased depth and leaflet edge definition.

Video 3 **(A)** Three-dimensional TTE zoom acquisition of the TV, as seen from the RV perspective. **(B)** TI rendering enhances border delineation as well as size and shape of the regurgitant orifice. *RV*, Right ventricle*.*

Video 4 **(A)** Conventional 3D zoom acquisition and **(B)** TI rendering on a patient with perforation of the AV NCC.

Video 5 Transillumination rendering of a stenotic MV, as seen from the LA (*left image*) and from the LV (*right image*). *LV*, Left ventricle.

Video 6 **(A)** Mitral valve P2 flail seen in 2D-TTE imaging **(B)** TI rendering provides enhanced definition of the affected MV scallop, the ruptured chordae, and the location and size of the defect.

## References

[1] Italiano G., Fusini L., Mantegazza V., Tamborini G., Muratori M., Ghulam Ali S. (2021). Novelties in 3D transthoracic echocardiography. J Clin Med.

[2] Genovese D., Addetia K., Kebed K., Kruse E., Yamat M., Narang A. (2019). First clinical experience with 3-dimensional echocardiographic transillumination rendering. JACC Cardiovasc Imaging.

[3] Karagodin I., Yamat M., Dow A., Rivera L., Singh A., Addetia K. (2022). Utility of transillumination and transparency renderings in 3D transthoracic imaging. Int J Cardiovasc Imaging.

[4] Hahn R., Weckbach L., Noack T., Hamid N., Kitamura M., Bae R. (2021). Proposal for a standard echocardiographic tricuspid valve nomenclature. J Am Coll Cardiol Imaging.

[5] Barbeito-Caamaño C., Bouzas-Zubeldía B., Martín-Álvarez E., Souto-Cainzos B., Bouzas-Mosquera A. (2021). An unusual presentation of prosthetic valve endocarditis: utility of 3D transillumination rendering. Echocardiography.

[6] Karagodin I., Shah A.P., Lang R.M. (2020). Guided by the light-transillumination of a paravalvular leak. JAMA Cardiol.

[7] Barreiro-Perez M., Cruz-González I., Martin-Moreiras J., Diaz-Pelaez E., Nuñez J.C., Luengo-Mondejar P. (2021). Transillumination and tissue-transparency photo-realistic echocardiography imaging during percutaneous mitral valve interventions. JACC Cardiovasc Interv.

